# Predicting Future Prospects of Aptamers in Field-Effect Transistor Biosensors

**DOI:** 10.3390/molecules25030680

**Published:** 2020-02-05

**Authors:** Cao-An Vu, Wen-Yih Chen

**Affiliations:** Department of Chemical and Materials Engineering, National Central University, Jhongli 320, Taiwan; vucaoan@gmail.com

**Keywords:** aptamer, small molecule, FET aptasensor, field-effect transistor, structure-switching, bio-amplifier

## Abstract

Aptamers, in sensing technology, are famous for their role as receptors in versatile applications due to their high specificity and selectivity to a wide range of targets including proteins, small molecules, oligonucleotides, metal ions, viruses, and cells. The outburst of field-effect transistors provides a label-free detection and ultra-sensitive technique with significantly improved results in terms of detection of substances. However, their combination in this field is challenged by several factors. Recent advances in the discovery of aptamers and studies of Field-Effect Transistor (FET) aptasensors overcome these limitations and potentially expand the dominance of aptamers in the biosensor market.

## 1. Introduction

One and a half century since the discovery of nucleic acid by Meister in 1869 [1], the properties and functions of aptamers have attracted significant research attention. The outburst of studies on this biomolecule began in the mid-20th century and continues to the present day. Aptamers are now among the most crucial nucleic acid species with versatile applications in separation, analytics, and molecular imaging, particularly in diagnosis and therapeutics. This perspective provides a prediction about the future development of aptamers in Field-Effect Transitor (FET)-based biosensors, a narrow field of sensing technology. To this end, this paper outlines a brief history of aptamers and, specifically, their function in targeting small molecules. We then follow with a summary about the key characteristics of FET and the major role of aptamers in sensing techniques employing this transducer. The content of these three sections foreshadows our final view on the matter as expressed in the final part.

## 2. Aptamer Timeline

Aptamers, simultaneously introduced in 1990 by two laboratories [2,3], are short single-stranded DNA or RNA that specifically bind various molecules with high affinity [4]. Both groups are considered to have independently contributed to the debut of aptamers because they employed a similar process to synthesize the biomolecule (but differently named it: SELEX by Lary Gold and Craig Tuerk, in vitro selection by Ellington and Szostak) [2,3]. Since their inventions, aptamers have received tremendous attention because of their desirable properties: they can be easily synthesized via a reversible SELEX process or a low-cost phosphoramidite; they have stable bioactivities within a wide range of thermal conditions [5,6,7,8]; it is feasible to create them without animal or cell line; there is no limitation to physiological environment of optimal functionality; they are easier to conjugate with chemical and biological molecules; and they have higher thermal stability and minimized immunological effects in comparison to antibodies and their fragments [5,6,7,8,9,10,11,12,13]. However, despite possessing several advantageous properties, two primary limitations of aptamers are their high-cost, tediousness, repetitiveness, the length of time consumed using the SELEX technique [14,15], as well as their vulnerability to nucleases, especially the RNA aptamer which poses a threat to ex vivo and in vivo applications. Other minor challenges include a dearth of accommodation for modified and unnatural nucleic acids due to the limited abilities of the polymerases [15,16,17] and the tendency of studies to design novel strategies instead of isolating new aptamers [11,18].

Regarding SELEX (or in vitro selection), the technique basically involves four primary steps: (1) generate a vast randomized library of nucleic acids which can be amplified by polymerase chain reaction (PCR); (2) incubate the library with targets of interests and separate the oligonucleotides specifically binding to the target from the unbound ones; (3) collect bound sequences and amplify them by PCR (this step is repeated to obtain a collection of sequences with high affinity to the target); and (4) clone and sequence the library. The evolution of the SELEX process has resulted in new generations which have overcome a number of the aforementioned drawbacks of aptamers [19]. Achievements have been reached in producing a simple, efficient, low-cost (Open qPCR SELEX) [20] and non-tedious SELEX process (High-Throughput Sequencing (HTS-SELEX)) [21] which shortens selection time (Capillary Electrophoresis (CE-SELEX) [22], Equilibrium Capillary Electrophoresis of Equilibrium Mixtures (ECEEM-SELEX) [23], FluMag-SELEX [9], Non-SELEX [24], MonoLEX [12], NanoSelection (nM-AFM SELEX) [25], Rapid-SELEX [26], Hi-Fi SELEX [27]). In addition, the stability of aptamers against nucleases can be improved via various chemical modifications (Spiegelmer) [16]. 

The vast majority of developments in the field of aptamers involve analytical techniques which rely on high affinity and specificity binding between aptamers and their coupling agents [28,29]. During the 30-year timeline of aptamers, those which direct proteins have received the most extensive efforts and resources for inventions and innovations. Lots of aptamers have been generated from their corresponding proteins: T4 DNA polymerase (RNA) [3], GCN4 protein of yeast (double-stranded DNA) [30], bacteriophage R17 coat protein (RNA) [31], E.coli functional proteins (phenylalanyl-tRNA synthetase, elongation factor-Tu, ribosomes and ribosomal protein) [32,33,34], U1snRNP-A (U1-A) protein [35], human *elav*-like neuronal protein 1 [36], basic Fibroblast Growth Factor (bFGF) [37], Tax protein of human T-cell lymphotropic virus (HTLV-I) [38], reverse transcriptase of Avian Myeloblastosis virus (AMV) and Moloney murine leukemia virus (M-MLV) [39]. 

Aptamers to thrombin and proteins encoded by the Human Immunodeficiency Virus (HIV) are the most remarkable. First introduced in 1992 by Bock ‘s group, 15-mer DNA aptamer can bind to human α-thrombin via fibrinogen-recognition exosite by forming a stable intramolecular G-quadruplex structure (chair-like conformation) [40]. Not so long later was the revelation of a longer DNA aptamer (29-mer DNA) binding to the heparin-recognition exosite by Tasset ‘s group [41]. This group also synthesized two RNA thrombin aptamers with 24 and 33 nucleotides of sequence length [42]. Meanwhile, aptamers that can select various proteins of HIV type 1 (HIV-1) including Rev protein [43,44], reverse transcriptase (RT) [43,45], and Tat [43] were recognized for their potential in therapeutics. Many of them have been continuously developed to inhibit the RT protein of HIV [46,47,48,49,50,51,52]. In a battle to replace antibodies destroyed by the virus, the spotlight gradually shifted to the diagnostic and therapeutic functions of aptamers in targeting immune systems [53,54]. A review of the attainments within this field was listed in Hu ‘s publication [55].

## 3. Aptamers Targeting Small Molecules

Aptamer binding of small molecules is another area which has consumed much effort and time of scientists and researchers. This field has attracted interest mainly due to the functions of small molecules, which are ubiquitous in a wide range of antibiotics, steroids, amino acids, nucleotides and its derivatives and miscellaneous organic ligands. On one hand, these molecules are beneficial in serving as research tools, drugs in therapeutics, pesticides in farming, secondary metabolites and neurotransmitters. On the other hand, they are detrimental, causing pollutions, cancers and abnormalities (teratogens). Appearing right after the disclosure of aptamer, aptamer–small molecule complexes have rapidly demonstrated their importance. The first decade witnessed a clustering of aptamers targeting amino acids (L-valine [56], L-isoleucine [57], D-tryptophan [58], L-arginine and L-citrullin [59]), antibiotics (Neomycin B [60], Tobramycin [61], Streptomycin [62], Viomycin [63], Kanamycin A and Lividomycin [64]) nucleotides and biological factors (Guanosine [65], Vitamin B12 [66], Xanthine [67], Flavin Adenine Dinucleotide and Flavin Mononucleotide [68], Nicotinamide Adenine Dinucleotide and Nicotinamide Mononucleotide [68]), miscellaneous organic ligands (dopamine [69], sulforhodamine B [70], Theophylline [71] and a variety of organic dyes [2]). However, the binding affinity between the aptamer sequences and their corresponding targets was relatively weak, with most of the *K_d_* value ranging from the hundreds of nanomolar to milimolar range. RNA dominated during this period because its libraries are considered a better choice for selection due to what many recognize as more diversified functional structures and higher affinity binders compared to DNA [4,72]. 

Since then, aptamers specifying these small substances have been increasingly retained and are recognized for their potential applicability in various fields. Structural studies of their complexes with corresponding targets have also enriched the research of tertiary motifs in RNA folding by contributing new motifs [73]. However, despite an encouraging beginning to aptamer research and experimentation, there was no significant outburst of aptamers which matched small molecules. This was largely due to the size difference between aptamers and small molecules, the challenges in setting up universal schemes for screening and characterizing, as well as issues related to measuring the binding affinity between such kinds of substances and their corresponding aptamers [74]. As a result, in approximately more than two decades of existence, there were only less than 20 percent of aptamers being selected for small molecules, with a minority of them specifying practical targets [75]. In spite of these weaknesses, aptamers corresponding to small molecules have attracted profuse investments and are among thriving aptamers at the center of much research. A literature survey on more than 900 publications about sensing technology based on aptamers in the first decade of the new century found that aptamers of ATP, cocaine, and theophylline are the second, fifth and seventh, respectively, most frequently used [76]. Moreover, new findings in this field coupled with high demands for aptamers targeting small molecules in therapeutics, medicine, analytical biochemistry and sensing technology means that the application of aptamers to small molecules remains a significant area of research. 

The current decade in the aptamer era is characterized by the resurgence of aptamers fitting small molecules (17β-estradiol [77], anatoxin-a [78], brevetoxin-2 [79], bromacil [80], danofloxacin [81], digoxin [82], oxytetracycline [83], quinolone [84], sphingosine-1-phosphate [85], T-2 toxin [86], thiazole orange [87] and zearalenone [88]) with significantly improved binding affinity (*K_d_* value at nanomolar scale) and the replacement of RNA by DNA. The rise and prevalence of DNA sequences in this period is attributed to their chemical and biological stability, low-cost and time-saving synthesis, making DNA aptamers more commercially favorable in comparison to RNA types [4]. Simultaneously, there have been numerous methods innovated to overcome the challenges and limitations of the screening and characterization of aptamers for small molecules [89,90,91,92,93]. A workflow diagram with four steps has been designed in an effort to optimally integrate aptamers for various applications [94]. Serving as a post-selection tool, the diagram aims to reduce the number of sequences at candidate screening; optimize and truncate binding sequences; determine K_D_ and other parameters for truncation and optimization; and assess aptamers for different usages. Detailed protocols of selection and amplification were constructed to isolate the aptamers for small-molecule sensors [95]. Intensive research on the fundamentals and principles of this area has attracted more investments [96,97,98] whilst binding affinity has also been strengthened at all cost [19,99]. Consequently, these breakthroughs have been accompanied by an expansion and improvement of the application of aptamers to small molecules in areas such as drug delivery [100] and molecular imaging [101], particularly in sensing technology where they are combined with electrochemistry, surface plasmon resonance, optical fibers, quantum dots, field-effect transistors, fluorescence and a variety of other techniques used to monitor contaminants, metal ions, hormones, explosives, toxins, drugs, antibiotics and pesticides [29,102,103,104,105,106,107,108]. With the emergence of the sensing small molecule technique, popular strategies to construct biosensors rely on protein-based receptors (antibodies and enzymes) [109]. Afterwards, aptamers, with the most essential advantage of high affinity and selectivity to these substances, unsurprisingly, have rapidly outdated these old-fashioned biomolecules to become the first priority in designing receptors for small molecule detections, especially during the past 5 years [29,102,103,104,105,106,107,108].

## 4. Importance of Aptamer in FET Biosensors

### 4.1. FET Biosensors: Working Principles and Limitations

In biosensor applications, FET is a three-electrode system that plays a role as a transducer which converts signals produced by bio-recognition events of the detected molecules and their receptors to electrical readout. The gate electrode is used to control the potential of bias. The bio-probes are immobilized onto the sensing channel that links source and drain electrodes to capture their targets, a process which varies the channel conductance. This variation is recorded and further processed by an electrical measurement system. FETs are classified into p-type and n-type based on doping methods and charge carriers. On one hand, in p-type devices, hole aggregation will increase the conductance if the negatively charged molecules are captured by the receptors. If the positively charged molecules are sensed, the conductance will decrease due to depletion of charge carriers (holes). On the other hand, in n-type devices, the recognition of positive charges raises the conductance due to electron accumulation, whilst binding negative charges results in conductance decline due to reduction of charge carriers (electrons).

In spite of ultra-high sensitivity and label-free detection, employing FET as transducers to produce biosensors for clinical diagnosis is hindered by physiological environments with high ionic strength and interferences from non-specific binding of numerous proteins in human serum and plasma. Both of these factors severely weaken the detected signal and deteriorate sensitivity of FET biosensors.

### 4.2. Aptamers: Sensing Elements Overcoming Limitations of FET Biosensors

Since FETs have been employed as transducers for biosensors, antibodies are the most widely applied recognition factor because of their simplicity, low-cost synthesis and ability to be immobilized on various surfaces. Particularly, antibodies can quickly detect their corresponding targets (antigens) with high affinity and specificity in so-called immunosensors, the most popular biomedical application of biosensors. However, since the bulky structure of the antibodies and the Debye-length poses a serious threat to clinical trials of many biosensors in physiological environments, a new generation, more compact probe (antibody fragments and aptamers) was born to surpass this obstacle. Aptamers have emerged as the most prominent sensing element in comparison to other antibody-derivative candidates for some of the reasons already mentioned in Section 2. More importantly, the compact structure of aptamers lets the target capturing happen under Debye length where electrical signal is detectable. This advantage will be discussed in more detail in the next paragraph in support of the main conclusions of this paper. 

## 5. Predicting Future Prospects of Aptamers in FET Biosensors

### 5.1. Aptamers as Bio-Receptors in FET Biosensors for Small Molecule Detection

Due to the rapid development of the field, the application of aptamers is productive territory for scientists to exploit, especially in the areas of diagnosis and therapeutics. Aptamers have been successfully applied in biosensing where they play roles as receptors and amplifiers. As a receptor, aptamers, in combination with various physical/chemical transducers, have triggered the development of a unique class of biosensors, so-called aptasensors, which possesses the dual function of detecting targets and producing signals from binding events [108]. The emergence of field-effect transistors has led to their integration with aptamers in order to detect a variety of molecules, most of which are macromolecules such as proteins, nucleic acids, and viruses [7,110,111]. 

In FET biosensor systems, aptamers are famous for replacing antibodies as bio-receptors (for the reasons mentioned in Section 1), as well as their compact structure which lets the target capturing happen under Debye length and allows electrical signal to be detected. FET biosensors have been used as a versatile tool for a wide range of molecular detection because they are one of the most powerful and ultra-sensitive transducers. Nevertheless, since the operation mechanism of FET relies on conductance change caused by charge variations on the sensing channel, recognizing binding events is only possible upon the detection of charged substances. Employing FET to detect capturing small molecules with low- or un-charged characteristics is therefore unpractical because variation from electrical signals produced by binding events between these targets and their probes is not strong enough, unstable and exhibits a low signal-to-noise ratio. 

Scientists in National Chiao Tong University are the pioneers who overcome these obstructions to detect a kind of uncharged molecule by FET. In their report, 19-norandrostendione (19-NA), a steroid, was recognized at femtomolar level by Δ^5^-3-ketosteroid isomerase (KSI), an engineered protein, on the silicon nanowire FET channel [112,113]. However, synthesizing KSI and chemically modifying it with a carbon chain-linked 5-(2-Aminoethylamino)-1-naphthalenesulfonic acid (1,5-EDANS) moiety was elaborate, complicated and required interdisciplinary knowledge. In addition, this process no longer made their techniques label-free. Subsequent years have witnessed a continuous development of FET sensors for tiny molecules with no/feeble charge and low molecular weight including trinitrotoluene [114], glucose [115,116], aflatoxin-B1 [117], zearalenone [117], ochratoxin-A [117]. Fundamental research on the interactions between small molecules and FETs has been the focus of recent scientific attention [118].

Not so long after the appearance of the FET sensor for 19-NA was the debut of an aptasensor which detects riboflavin via the integration of DNA aptamers specifying to this molecule and FET (Zinc Oxide - ZnO and Single-Walled Carbon Nanotube - SWCNT) [119,120]. In their report, Hagen et al. exploited conformational change of aptamers after binding riboflavin, carrying their negative-charged phosphodiester backbones closer to the surface of the FET channels and inducing signal variation. Detection of adenosine triphosphate (ATP), bisphenol A (BPA), and β-estradiol by FET aptasensors was also accomplished [121,122,123,124,125] (publications fabricating FET aptasensors to detect small molecules are listed in Table 1). More recently, Nakatsuka and his colleagues depended on a similar strategy for successfully sensing serotonin, dopamine, glucose and sphingosine-1-phospate (S1P) by stem-loop aptamers and FET in a physiological environment [126]. In fact, structure-switching is a familiar mechanism which is capable of converting the binding event into detectable signals, something that has already been proved in numerous aptasensors [127,128,129,130,131].

Considering these achievements, conformational changes after target binding, admittedly, have unique advantages over aptamer in sensing insignificant-charged molecules. Abundant negative charges along aptamers’ structure which approach the proximity of the FET surface obviously solve the problem of the low electrical signal created from binding events. In addition, binding events occurring on sensing channels of FET aptasensors are under Debye length and therefore can produce a consistent signal. Once the aforementioned issues have been solved, FET, exhibiting the strong points already demonstrated (such as being ultra-sensitive, label-free, low-cost and having the potential to be commercialized (SiNW)), will be capable of detecting entities at extremely low concentrations in serum samples. FET will then become a powerful tool for various biomedical applications. It is predicted that employing induced-change in conformation (structure-switching mechanisms) after detecting the target for FET biosensors will be a focal direction for aptamer applications in the future. This is particularly so considering rapid developments in the invention of aptamers targeting small molecules in recent years as well as promising progress in the design of FET aptasensors.

### 5.2. Aptamers as Bio-Amplifiers for FET Biosensors

Aptamers in biosensors also have a role as amplifiers, a crucial function which boosts the signal-to-noise ratio, particularly in ultra-sensitive assays detecting targets with trace amounts and/or operating conditions with high interference (physiological environment). Aptamers have thus been successfully exerted as bio-amplifiers or assisted in amplifying detection for various types of sensors [132]. In an FET biosensor system, a majority of noises comes from the intrinsic quality of the devices [133] (flicker noise from mobility of charged carriers [134,135], thermal noise relating to conductivity and density of charged carriers [135]), whilst other contribution factors are environmental fluctuations [136], interactions between biomolecules and the channel surface [137], substrate interactions and surface adsorbates [138,139]. Furthermore, in many biomedical applications of FET nanosensors in clinical diagnosis, electrical signals actuated from the binding event are drastically weakened by the ionic screening effect, also known as Debye screening, caused by the high ionic strength of the physiological environment (>100 mM) [140]. The screening effect strongly depends on the distribution of various charged groups (positive and negative) throughout the targets and their distances to the sensing surface [141,142]. Electrical change recorded in detecting oligonucleotides, especially microRNA with short sequences, is therefore attenuated and sensitivity is deteriorated. These effects are more severe in applications of FET nanosensors with protein assays where signal shift is inconsistent and unstable [142,143]. For these reasons, an amplifier is even more essential and inevitable for FET biosensors to detect substances at their ultra-low concentration in serum and/or plasma samples.

A number of properties intrinsic to aptamers are advantageous for amplifying the signal from sensing various molecules by FET. Such properties include the possession of various structural switching mechanisms from aptamer-target complexes which can facilitate the transduction of the signal from binding events; a high affinity and selectivity to a wide variety of entities which can prevent the cross-reaction with other substances in the assays; and low molecular weight and compact structure which feasibly let the binding event occur under the Debye length and allow signal variation to be detected. 

Possessing highly negative-charged backbones is the most essential and unique characteristic of aptamer which can facilitate signal enhancement of FET biosensors. Indeed, robust negative charged molecules have been successfully exploited for signal amplification of FET biosensors in several publications and have recently become more popular. The pioneer group of this creativity used the rolling circle amplification (RCA) method to produce repeated DNA sequences as amplifiers for their SiNW-FET HBA sensors. Abundant negative charges from elongated oligonucleotides on the nanowire surface significantly improved the signal-to-noise ratio of their fabricated FET biosensors by more than 20× to detect HBV DNA at a decreased concentration of 1 fM [144]. RCA was also used by Chen et al. to enhance sensitivity of aptameric extended-gate field-effect transistors (EGFETs) sensing platelet-derived growth factor (PDGF) [145]. A more complicated strategy, also based on the mechanism of negative charged DNA, was published by a group of scientists from the United States, China, Hong Kong and Ecuador. In their report, a copious amount of negative charges was created via hybridization chain reaction (HCR) and by the assistance of various DNA helpers. Amplified products from target recycling and HCR remarkably boosted the detection limit of their fabricated GFET nucleic acid sensors down to 5 fM, which is 20,000× lower in comparison to similar studies with single-stranded DNA and complementary hybridization [146]. 

The simplest amplification technique based on negative charges came from Osaka and his colleagues who introduced sodium dodecyl sulfate (SDS), an ionic surfactant which can couple with buckwheat protein 16 (BWp16) to provide an amplifier for their FET sensors to detect this allergenic target [147,148]. Most recently, Chen and his teammates succeeded in proving the concept of aptamer as bio-amplifier for both direct and sandwich immunoassay by SiNW-FET [143]. The application of aptamers as bio-amplifiers for FET-based biosensors has therefore become a very promising prospect. Moreover, early developments in the field which demonstrate the ability of aptamers to specifically bind with different biomolecules (thrombin, HIV proteins), as well as recent advances in aptamer targeting of small molecules, means that aptamers can potentially contribute to FET sensing technology not only as bio-probes but also as bio-amplifiers in a so-called sandwich FET aptasensors.

In such familiar architectures which have already prospered in several platforms [128,149,150,151,152], primary aptamers can be immobilized on FET channels to detect analytes (proteins, DNA, RNA, small substances, low-/un-charged molecules) before coupling with secondary aptamers for signal enhancement. This scheme potentially unblocks another approach for sensing heavy metal ions constituting a huge proportion of the biosensor market [105,153,154,155]. In summary, the continuous growth of selection technology for aptamer binding of small molecules, as well as the promising potential of FET, is likely to sustain interest in fulfilling the demands of the biosensor market.

## Figures and Tables

**Table 1:** Development of FET aptasensors detecting small molecules.

Field-Effect Transistors	Bio-Receptors	Target Molecules	Ref.
SWCNT	Riboflavin-binding Aptamer	Riboflavin	119
ZnO	Riboflavin-binding Aptamer	Riboflavin	119, 120
SWCNT	Anti-BPA Aptamer	BPA	121
SWCNT	ssDNA anti-ATP Aptamer	ATP	122
CNT Network Film	35-mer E2 Aptamer	17 β-estradiol (E2)	123
Graphene	Aptamer	ATP	124
Carboxyl-functionalized Multichannel Carbon Nanofibers	BPA-binding Aptamer	BPA	125
Indium (III) Oxide	Dopamine Aptamer Serotonin Aptamer S1P Aptamer Glucose Aptamer	Dopamine Serotonin S1P Glucose	126
